# A national snapshot of family caregivers in the United States: characterizing demographics and health among caregivers over age 50

**DOI:** 10.3389/fpubh.2026.1886926

**Published:** 2026-08-24

**Authors:** Hua Zan, Loriena A. Yancura, Melissa A. Barnett

**Affiliations:** 1 Center on the Family, University of Hawaii at Manoa, Honolulu, HI, United States; 2 Department of Family and Consumer Sciences, University of Hawaii at Manoa, Honolulu, HI, United States; 3 Human Development and Family Science, The University of Arizona, Tucson, AZ, United States

**Keywords:** Health and Retirement Study, intergenerational caregiving, middle-aged and older adults, multi-generational caregivers, single caregivers

## Abstract

**Introduction:**

As modern households diversify, many family caregivers face multi-generational demands, such as simultaneously caring for grandchildren and aging parents. Despite being the backbone of the care system, caregivers navigate highly varied needs and family dynamics. It is thus crucial to understand who family caregivers are and how they fare in terms of health and wellbeing. This descriptive study provides a profile of U.S. intergenerational caregivers over age 50.

**Methods:**

Using data from 2022 RAND Health and Retirement Study (HRS) and HRS core files, we focus on four primary caregiver types—parent caregivers, spousal caregivers, parents of minor children, and grandchild caregivers. We first estimate the percentage of 15 total caregiver configurations, including those caring for single or multiple categories of family members. We then describe and compare the sociodemographic characteristics across the four primary caregiver types, looking specifically at those caring for parents only, spouses only, children only, or grandchildren only. We also compare self-reported health indicators of these same four groups caring for one generation only after adjusting for age differences.

**Results:**

Approximately 85% of caregivers provided care solely to one generation of care recipients (i.e., parents, spouses, children, or grandchildren), while the remaining 15% provided simultaneous care for multiple generations of recipients. Sociodemographic and health indicators varied by specific caregiver types. Spousal caregivers had worse self-reported health and higher depression scores compared to other caregiver types, even adjusting for age differences.

**Discussion:**

While family caregiving is common among adults over 50, their demographics and experiences vary significantly. This study underscores a critical need to move beyond a “one-size-fits all” support model. Notably, spousal caregivers remain uniquely vulnerable, requiring targeted clinical interventions. Additionally, the prevalence of multi-generational caregiving requires policy expansions—such as inclusive leave policies—to address overlapping role strains.

## 1 Introduction

Unpaid, informal family caregivers are the bedrock of household support and a cornerstone of the American economy. The landscape of family caregiving in the United States has changed dramatically over the past few decades (1). Life expectancy has increased (2) while fertility has decreased, leading to a greater number of living generations (3). Growing complexities in family structure have led to families with more individuals in need of care and fewer able to provide it (2, 4). Economic hardships and changes in government policies place greater caregiving responsibility on families (5), particularly middle generations (4, 6).

These demographic and sociopolitical changes have also led to diversification of *types* of family caregivers with potentially unique needs, yet we have limited knowledge regarding the prevalence of specific types of family caregivers. The term *family caregiver* encompasses a variety of care recipients and generations (i.e., parent, spouse, minor child, or grandchild). Each caregiver-recipient relationship involves unique tasks, responsibilities, skills, and family dynamics (7), yet scant research has focused on differences between these distinct caregiving types. For example, nurturing a minor child and providing health-related care vary significantly in both their primary focus and the needs they address. While caring for minor children emphasizes fostering healthy development through nurturing, responsive interactions (8), health-related and elder care centers on managing medical demands and daily functional support (9). Furthermore, variations persist even within older adult caregiving itself. Prior studies often lump together spousal caregivers (i.e., those providing care to spouses) and parent caregivers (i.e., those providing care to aging parents) (10), despite the potential for different influences in wellbeing of each caregiver type (11).

This study distinguishes between caregiving types based upon care recipients’ generation relative to the caregiver and describes the prevalence of caregiving type among family caregivers over 50 in the U.S. We also identify and compare demographic characteristics among four primary caregiver types (i.e., caring for parents only, spouses only, children only, and grandchildren only) and compare key health indicators across the four primary caregiver types after adjusting for age differences. Drawing upon life course developmental theory, this descriptive study uncovers differences in risk factors, protective factors, and wellbeing across types of family caregivers. The resulting nuanced understanding of care experiences is highly relevant for designing and implementing targeted programs that meet the unique needs of caregivers and their family members.

### 1.1 Life course developmental framework

This study is guided by the life course developmental framework (12, 13). From this perspective, caregiving is a dynamic, relational process deeply embedded in family systems and generational interdependence (14) and a continual negotiation of roles that structurally, functionally, and emotionally interlocks the wellbeing of multiple generations (15). The life course developmental framework focuses on three critical and interlinked factors. First, individuals within family networks share histories that shape relationships in unique ways over time (13). For example, caregivers may consider past experiences of being cared for by care recipients while they perform caregiving tasks or make decisions. Second, family networks are composed of generations whose relationships may change over time (12). This shift can change power dynamics (e.g., between couples), which then influence the caregiver’s wellbeing. Third, the developmental framework highlights the influence of the current developmental stages of each member of the caregiving dyad on their interactions and wellbeing (13). While family caregiving is associated with physical and emotional demands (16), the specific intergenerational context influences the impact of these demands (3, 17).

Applied to health outcomes, this framework suggests several mechanisms linking caregiver type to wellbeing: direction of care, expectation, and motivation. First, the direction of care may be associated with distinct meanings of the caregiving role. Caregiving for younger generations might represent generativity or concern for the future (18), in contrast to caregiving for older generations, which might be associated with loss and death (19). Second, expectations can shape role adaptation. Caring for an aging parent represents an anticipated life-course transition that allows for psychological preparation, whereas spousal caregiving is often sudden, disrupting marital dynamics and causing strain (13, 20). Third, motivations for care provision can vary across caregiving types. Parent and grandchild caregiving is often motivated by reciprocity, whereas caring for ill spouses or minor children is an obligation. Consequently, mandatory roles risk draining mental strength, while volitional, generative roles may offer protection for health. Ultimately, caregiving experiences vary considerably because it is embedded in lifelong relationship dynamics, expectations, and interconnected experiences.

### 1.2 Types of family caregivers

#### 1.2.1 Parent caregivers

According to AARP and the National Alliance for Caregiving (21), 60% of family caregivers aged 50–64 care for a parent, parent in-law, or spouse. Family caregiver duties range from sporadic care (e.g., light errands, accompanying to medical appointments) to coordination of in home or institutional care, and eventually helping the care recipient prepare for death and managing their affairs after death (22).

Thus, the impact of caregiving on caregiver wellbeing is individualized and varies depending upon care recipient, lifelong intergenerational relationship experiences, as well as familial, social, and economic contexts (22). In general, caregivers for older adults score higher on measures of depression, stress, and self-efficacy, and lower on measures of subjective wellbeing, than their non-caregiving peers (10). Caregivers for recipients with dementia (10), those providing care for a greater number of hours (23), and those involved in family conflicts around care score lower on measures of physical and mental health than those with “lighter” caregiving loads (17).

#### 1.2.2 Spousal caregivers

Individuals who provide care to a spouse are often included in studies of caregivers for older adults without differentiating between spousal and parental care (24). However, spousal caregivers are typically older and less likely to have paid employment than those caring for aging parents (25). Additionally, these groups appear to differ on the types of caregiving stressors they experience and their subjective perceptions of caregiving burden (26). More work is needed to explore between-group differences.

#### 1.2.3 Parents of minor children

Although parenting is not generally considered in broader definitions of family caregiving (19, 22), the life course developmental framework suggests considering parent-minor child relationships because these intergenerational relationships change over time in ways that influence and are influenced by shifts in each generation. Parenting a minor child after age 50 presents unique challenges, including diminished social support and heightened concerns about mortality (27). Parenting a minor during mid-life and beyond may be misaligned with expectations for family responsibilities. In general, older parents report high levels of stress and life satisfaction (28). There is little research that compares health outcomes associated with caring for older adults and parenting minor children among similar-aged adults. As the mean age of parenthood in the U.S. has risen in recent years (29), understanding the wellbeing of adults over 50 who are caring for minor children is of increasing importance.

#### 1.2.4 Grandchild caregivers

Grandparents are increasingly involved in caring for grandchildren, ranging from occasional babysitting to serving as primary or custodial caregivers (30). The number of grandparent caregivers who provide supplemental care alongside parents in the U.S. is difficult to estimate. One study reported that nearly 60% of U.S. grandparents live within an hour’s drive of their grandchildren. Of those, 40% with grandchildren under 14 years old provide unpaid childcare for their working children (31). Due to the high costs and limited accessibility of childcare, four out of five working parents report relying on grandparents for at least some childcare (32). The 2026 AAPR grandparenting report found 10% of grandparents aged 35 or older provide primary care for grandchildren with full custodial responsibilities (33). Grandparents raising grandchildren often provide unpaid care driven by a sense of moral responsibility (34, 35). Caring for grandchildren is inherently influenced by three-generation family life course dynamics that may make this experience distinct from other family caregiving arrangements.

### 1.3 Age and health indicators

Age is a critical factor in caregiving research because it intertwines with roles and health outcomes. Chronological age is intrinsic to human development and, to a lesser extent, defines developmental stage (36). Its influence is further compounded by overlapping social and contextual factors (37) that may jointly influence caregiver outcomes. For example, compared to younger counterparts, older adults are more likely to be retired, rely on fixed sources of income, and live in rural than urban areas (38). Advanced age is generally associated with higher levels of chronic disease (39), and lower levels of psychological stress (40).

Examining health indicators in adults over 50 is important due to potential associations with unique strains of each caregiving type. For example, changes in self-rated health or Body Mass Index (BMI) might influence the decision to take on family caregiving roles, and be a direct or indirect product of providing care to older adults such as spouses or parents (22, 41). Depression is frequently associated with family caregiving, but also is correlated with situational factors such as isolation (42). Research also indicates, while spousal caregiving can increase the likelihood of moderate-to-vigorous physical activity (43), grandparents providing intense childcare engage in less physical activity (44).

### 1.4 The current study

This study estimates the prevalence of distinct caregiver types among U.S. adults over 50 and constructs basic profiles of four primary caregiver types who care for *only* one category of family member: parents, spouses, children, or grandchildren. Specifically, we aim to first estimate the percentage of 15 total caregiver configurations including single caregivers (i.e., caregivers for only one category of family member) and those caring for multiple categories of family members (i.e., combinations of the four primary types listed above). We then describe and compare the sociodemographic characteristics of the four primary caregiver types. Additionally, we compare health indicators of these four primary caregivers after adjusting for age differences.

## 2 Materials and methods

### 2.1 Data and sample

This study used data from 2022 RAND Health and Retirement Study (HRS) family files and HRS core public files. The HRS is sponsored by the National Institute on Aging (grant numbers NIA U01AG009740 and NIA R01AG073289) and conducted by the University of Michigan. This longitudinal study collects information from a representative sample of approximately 20,000 people over age 50 in America. The RAND HRS data are a user-friendly version of the HRS public data created by researchers at the RAND Corporation. RAND HRS Family Data includes variables about respondents’ families such as characteristics of children, children-in-law, and grandchildren of HRS respondents and spouses (45). RAND HRS Longitudinal File covers much of the public data of the HRS Core and Exit interview on the respondent- and family-level characteristics such as demographics, health, and total income (46). We chose 2022 data because it is the most recent available HRS data at the time of the analysis.

We identified individuals caring for parents, children, and grandchildren from the Family Data, extracted their characteristics from the Longitudinal File, and merged the two data files. Because RAND HRS data include a subset of the original HRS data, we extracted spousal caregiver information from the HRS core public files and merged it with our dataset.

In our sample of 15,856 adults, 10,954 (69.1%) were not caring for parents, spouses, children, or grandchildren, whereas the remaining 4,902 (30.9%) individuals were caregivers.^1^ Among these caregivers, 4,110 individuals were caring for one type of family members (single caregivers), 741 were dual caregivers (e.g., caring for both parents and grandchildren), and 50 were caring for three or four types of family members.

### 2.2 Measures

#### 2.2.1 Caregiving type

We focused on four primary caregiving types: (1) parent caregivers; (2) spousal caregivers; (3) parents of minor children; and (4) grandchild caregivers. *Parent caregivers* were those who provided more than zero hours helping their own or spouse’s father, mother or both parents with basic personal needs such as dressing, eating and bathing, or with errands, household chores and transportation since the last interview. *Spousal caregivers* were identified using an indirect method because HRS does not directly ask whether respondents provide care to their spouses. Following the methodology of Choi et al. (47) and Lee and Tang (48), we identified spouses as caregivers if spouses were reported as the primary or secondary caregiver for respondents who had difficulties in activities of daily living (ADLs) or instrumental activities of daily living (IADLs). It is important to note, due to our extraction method, those identified as spousal caregivers tended to be those who provided care for individuals with relatively severe health problems. *Parents with minor children* were those with children under 18 years old. *Grandchild caregivers* were those providing more than zero hours of childcare for grandchildren or great-grandchildren since the last interview. Because some individuals provided care for multiple family members, we identified a total of 15 distinct caregiver configurations.

#### 2.2.2 Health indicators

We examined four health indicators: (1) self-reported good health; (2) BMI; (3) depression; (4) physical activity. *Self-reported good health* was coded dichotomously as ‘1’ for excellent, very good, or good health or ‘0’ for fair or poor health. *BMI* was calculated based on self- or proxy^2^-reported weight and height and using weight divided by height squared. *Depression* was measured with the Center for Epidemiologic Studies Depression (CESD) scale that includes six “negative” indicators (e.g., felt alone, felt sad) and two reverse-coded “positive” indicators (e.g., felt happy). The HRS defines *physical activity* as the frequency of engaging in vigorous, moderate, or light activity (daily, >1 per week, 1 per week, 1–3 per month, or never). Because research has established the health benefits of moderate-to-vigorous physical activity (MVPA) and its greater impact on reducing mortality risk compared to light activity in older adults (49, 50), we created a dichotomous variable indicating whether the respondent engaged in MVPA daily.

#### 2.2.3 Sociodemographic and economic characteristics

We considered age, sex, race/ethnicity (non-Hispanic White, non-Hispanic Black, Hispanic, or other), educational attainment (less than high school, high school graduates, some college, or college graduate or more), employment status (employed, unemployed, retired, disabled or not in the labor force), total income, and total assets. For the categorical variables (e.g., race/ethnicity), missing values were treated as an explicit category. For continuous variables (e.g., BMI), we evaluated the extent of missingness by calculating the percentage of missing data. Overall, the missingness in the covariates is minimal, ranging from 0.15–5.13% among single caregivers.

### 2.3 Analytical approach

We first estimated the prevalence of different types of caregivers. Subsequently, we focused on those caring for parents only, spouses only, children only, and grandchildren only. We compared the sociodemographic characteristics across these four groups using *F*-tests. We then compared age-adjusted health indicators—self-reported being in excellent/very good/good health, BMI, depression, and engaging in MVPA daily—across the four caregiving types. To adjust for age differences, we used logistic regression models for dichotomous health indicators (i.e., self-reported being in excellence/very good/good health, and daily engagement in MVPA) and ordinary least square regression models for continuous variables (i.e., BMI and depression scores). Caregiver type and age were the only covariates included in each model. We then calculated the average predicted probabilities or means of health indicators for each caregiver type followed by pairwise comparisons between groups using Bonferroni’s method (51).

To account for the complex, multi-stage probability sampling design of the HRS, all analyses were conducted using the survey estimation (*svy*) suite in Stata, incorporating the person-level sampling weights, primary sampling units, and stratification variables to ensure population representativeness and valid variance estimation.

## 3 Results

### 3.1 Caregiver types

Table 1 presents the weighted percentages of different types of caregivers over age 50 in the U.S. The vast majority of the caregivers (85.1%) cared for only one type of family member (single caregivers). A smaller but still noteworthy portion were dual caregivers (14.3%), and a few (0.6%) cared for three or more types of family members. Among single caregivers, over a third (33.6%) were parent caregivers, and nearly one third (32.0%) were grandchild caregivers. Furthermore, about 6.8% were parents of minor children, and 12.7% spousal caregivers. For dual caregivers, those caring for both parents and grandchildren were most prevalent (7.8%). In contrast, other dual caregiver combinations were less common: parents and children (2.72%), spouses and grandchildren (1.6%), parents and spouses (1.4%), and spouses and children (0.1%). While the “sandwich generation” caregiving traditionally refers to the simultaneous care for aging parents and minor children (52, 53), our estimates revealed that non-traditional “sandwiches” configuration—caring for parents and grandchildren—is more prevalent than the traditional role. Although much less prevalent, some individuals cared for three or more types of family members. The largest group in this category simultaneously cared for parents, children and grandchildren (0.3%).

**Table 1:** Weighted percentages by type of caregiver, 2022 Health and Retirement Study.

Type of caregiver	%
Single caregivers (caring for one type of family member)	85.08%
Parent caregivers only	33.60%
Spousal caregivers only	12.70%
Parents of minor children only	6.78%
Grandchild caregivers only	32.00%
Dual caregivers	14.33%
Parent + spouse	1.41%
Parent + child	2.72%
Parent + grandchild	7.83%
Spouse + child	0.14%
Spouse + grandchild	1.58%
Child + grandchild	0.65%
Caring for 3 types of family members	0.60%
Parent + spouse + child	0.08%
Parent + spouse + grandchild	0.23%
Parent + child + grandchild	0.27%
Spouse + child + grandchild	0.02%

“Child” refers to minor children. The “Parent + child” dual caregiver category corresponds to traditional “sandwich generation” caregivers.

### 3.2 Caregiver characteristics

Table 2 presents the weighted sociodemographic characteristics of the four primary caregiving types among single caregivers. The average age of older *parent caregivers* was 63.5 years and over half were female. About 71% were non-Hispanic White, 9.9% were non-Hispanic Black, 11.2% were Hispanic, and 7.6% were in the other racial/ethnic group. In terms of educational attainment, 4.8% had less than high school education, 26.2% were high school graduates, 31.9% had some college, and 37.1% were college graduates or had higher education. The majority of these caregivers (71.8%) were married or partnered. Approximately 40.8% were employed, and 51.3% were retired. Eight in 10 reported excellent, very good, or good health. The average depression score was 1.3, and BMI was 29.4. Only 19.6% engaged in MVPA daily. The total income was $132,888 and the total asset was $1,056,721 on average.

**Table 2:** Weighted sociodemographic characteristics of single caregivers by caregiving types with group comparisons, 2022 Health and Retirement Study.

Sociodemographic characteristics	Parent caregivers only	Spouse caregivers only	Parents of minor children only	Grandchild caregivers only	*p*	Cohen’s *d*
*N*	1,580	628	667	1,235		
Respondents’ characteristics						
Age	63.51 (4.68)	71.88 (9.30)	61.70 (5.80)	67.07 (5.81)	***	1.677
Female	52.50%	48.70%	25.10%	54.90%	***	0.200
Race/ethnicity					***	
Non-Hispanic white	71.00%	70.30%	51.30%	79.60%		0.014
Non-Hispanic black	9.90%	8.20%	17.50%	8.00%		0.031
Hispanics	11.20%	17.50%	19.70%	7.20%		0.320
Other	7.60%	3.90%	11.30%	5.20%		0.209
Missing (*n* = 211, 5.13%)	0.30%	0.00%	0.10%	0.00%		
Educational attainment					***	
Less than high school	4.80%	18.10%	18.30%	6.00%		0.287
High school graduates	26.20%	35.50%	17.90%	25.50%		0.208
Some college	31.90%	27.30%	29.10%	30.00%		0.128
College graduate or more	37.10%	19.10%	34.80%	38.60%		0.276
Marital status					***	
Married/partnered	71.80%	100.00%	68.50%	79.30%		0.068
Separated/divorced	14.30%	0.00%	24.60%	10.20%		0.164
Widowed	3.60%	0.00%	3.40%	8.40%		0.306
Never married	10.10%	0.00%	2.90%	2.10%		0.274
Missing (*n* = 6, 0.15%)	0.20%	0.00%	0.60%	0.10%		
Employment status					***	
Employed	40.80%	14.20%	52.90%	28.40%		1.066
Unemployed	2.90%	1.30%	2.80%	2.00%		0.056
Retired	51.30%	78.30%	36.40%	67.30%		1.241
Disabled or not in the labor force	4.90%	6.20%	7.90%	2.30%		0.372
Health indicators						
Self-reported health					***	
Excellent	8.00%	5.90%	9.30%	6.60%		0.240
Very good	39.20%	24.60%	24.20%	44.60%		0.149
Good	36.10%	34.20%	35.20%	33.60%		0.012
Fair	12.90%	27.20%	23.70%	12.20%		0.236
Poor	3.70%	7.70%	7.50%	2.90%		0.197
Missing (*n* = 6, 0.15%)	0.10%	0.30%	0.20%	0.00%		
Depression (max = 8)	1.27 (1.64)	1.61 (2.14)	1.32 (2.02)	1.06 (1.67)	***	0.127
BMI	29.35 (5.42)	28.63 (6.62)	29.55 (7.75)	29.09 (5.35)		0.144
Missing (*n* = 23, 0.56%)	0.00%	0.50%	1.50%	0.10%		
Engaging in moderate-to-vigorous physical activity everyday	19.60%	13.60%	14.70%	14.50%		0.090
Missing (*n* = 20, 0.49%)	0.30%	0.30%	2.00%	0.40%		
Household characteristics						
Total income	132,888 (212,164)	69,168 (90,186)	130,952 (188,744)	120,820 (123,105)	***	0.295
Total asset	1,056,721 (1,493,151)	605,530 (1,285,020)	1,442,660 (4,250,835)	1,163,077 (1,805,397)	***	0.092

Standard deviation reported in brackets. Statistical significance tests regarding differences in marital status were restricted to parent caregivers, parents of minor children, and grandchild caregivers. Cohen’s *d* was calculated to estimate effect size of the difference between the highest and lowest group means. *p < 0.1, **p < 0.05, ***p < 0.01.

The average age of *spouse caregivers* was 71.9 years and 48.7% were female. About 70.3% were non-Hispanic White, 8.2% were non-Hispanic Black, 17.5% were Hispanic, and 3.9% were in the other racial/ethnic group. In terms of educational attainment, 18.1% had less than high school education, 35.5% were high school graduates, 27.3% had some college, and 19.1% had a college degree or higher education. The majority (78.3%) were retired. About 64.7% reported excellent, very good, or good health. The average score for depression was 1.6, and BMI was 28.6. Only 13.6% engaged in MVPA daily. The total income was $69,168, and the total asset was $605,530 on average.

The average age for *parents with minor children* was 61.7 years; about one quarter were female. About 51.3% were non-Hispanic White, 17.5% were non-Hispanic Black, 19.7% were Hispanic, and 11.3% were in the other racial/ethnic group. In terms of educational attainment, 18.3% had less than high school education, 17.9% were high school graduates, 29.1% had some college, and 34.8% graduated from college or had higher education. The majority were married or partnered (68.5%). About 52.9% were employed, and 36.4% were retired. About 68.7% reported excellent, very good, or good health. Their average score for depression was 1.3, and BMI was 29.6. Only 14.7% engaged in MVPA daily. Their average total household income was $130,952, and the average asset was $1,442,660.

The average age of *grandchild caregivers* was 67.1 years with 54.9% being female. About 79.6% were non-Hispanic White, 8% were non-Hispanic Black, 7.2% were Hispanic, and 5.2% were in the other racial/ethnic group. In terms of educational attainment, 6% had less than high school education, 25.5% were high school graduates, 30% had some college, and 38.6% were college graduates or had higher education. Approximately 79.3% were married or partnered. The majority (67.3%) were retired. About 84.8% reported excellent, very good, or good health. The average score for depression was 1.1, and BMI was 29.1. Only 14.5% in this category engaged in MVPA daily. The average household income was $120,820 and the average asset was $1,163,077.

Comparison across these four caregiving types revealed statistically significant differences in demographic characteristics, including age, sex, race/ethnicity, educational attainment, marital status, employment status, self-reported health, depression, and economic status. Specifically, spousal caregivers and grandchild caregivers tended to be older, whereas parents of minor children and parent caregivers were younger. About 54.9% of grandchild caregivers were female, whereas only 25.1% of parents of minor children were female. Parents of minor children were the most racially/ethnically diverse. While over half of parents of minor children were non-Hispanic White, this figure was over 70% for other caregiving types. In terms of educational attainment, only 19.1% of the spousal caregivers were college graduates or higher education. In contrast, over a third of other types of caregivers had this level of education. Spouse caregivers were excluded from the marital status comparison since they are all married. Among the remaining three groups, grandchild caregivers had the highest percentages of married individuals and lowest percentages of separated or divorced individuals. Conversely, parent caregivers had the highest percentages of never-married individuals. In terms of employment status, spousal caregivers and grandchild caregivers had higher percentages of being retired, whereas parents of minor children and parent caregivers had higher percentages of being employed. Spousal caregivers had the highest percentages of reporting being in fair or poor health, highest depression scores, and lowest percentages of engaging in MVPA. In terms of economic status, spousal caregivers are worse off with lower income and assets compared to other groups. To evaluate the magnitude of these differences, we calculated effect sizes (Cohen’s *d*) comparing the highest and lowest group means using Cohen’s (54) widely accepted thresholds of 0.20, 0.50, and 0.80 to represent small, medium, and large effects, respectively. While these differences are statistically significant, the observed effect sizes are largely small, except for large effects found for age and employment status (being employed or retired).

### 3.3 Caregiver health

Because age is a crucial factor influencing health and time allocation, we adjusted for age when comparing specific health indicators with weighted results presented in Table 3. After this adjustment, spouse caregivers remained the most vulnerable group, with the lowest self-reported health and the highest depression. In contrast, parent and grandchild caregivers were better off with higher percentages of reporting excellent, very good, or good health, and lower depression scores. Importantly, there was no significant difference across all four groups in BMI and the percentages of those engaging in MVPA daily.

**Table 3:** Age-adjusted weighted averages for health indicators by caregiver types, 2022 Health and Retirement Study.

Health indicators	A. Parent caregivers only	B. Spouse caregivers only	C. Parents of minor children only	D. Grandchild caregivers only
Self-reported in good health	0.836	0.639	0.694	0.847
BMI	29.006	29.006	28.949	29.246
Depression	1.218	1.746	1.224	1.081
Everyday MVPA	0.194	0.142	0.146	0.147

Comparison across caregiver types						
Health indicators	B vs. A	C vs. A	D vs. A	C vs. B	D vs. B	D vs. C
Self-reported in good health	***	***	n.s.	n.s.	***	***
BMI	n.s.	n.s.	n.s.	n.s.	n.s.	n.s.
Depression	***	n.s.	n.s.	n.s.	***	n.s.
Everyday MVPA	n.s.	n.s.	n.s.	n.s.	n.s.	n.s.

MVPA stands for moderate-to-vigorous physical activity. *p < 0.1, **p < 0.05, ***p < 0.01, n.s., not significant.

## 4 Discussion

We find 31% of the U.S. adults over age 50 engage in family caregiving, with sociodemographic characteristics and health indicators stratified by caregiving types. Notably, this figure likely underestimates the true prevalence in this age group as the data limitations prevented us from identifying caregivers for disabled adult children, siblings, and other relatives. These individuals were classified into the “non-caregiver” group in this study. Nevertheless, our findings support a life course developmental framework, confirming that caregiving populations cannot be analyzed as a single, homogenous group.

Specifically, spousal caregivers fared the worst across most metrics, even when age was controlled. This finding, however, should be interpreted with caution due to a methodological limitation. Spousal caregivers were identified as those helping a spouse with ADLs or IADLs. Consequently, this group tends to represent more intense caregiving scenarios. In contrast, other caregivers (caring for parents, children, and grandchildren)—identified by positive caregiving hours—encompassed a broader spectrum of intensity, ranging from light to severe. Because we cannot directly control for caregiving intensity across these definitions due to data limitation, this asymmetric inclusion criteria may plausibly account for the health disparities between caregiver types. Additionally, we controlled only for age. Differences in uncontrolled factors such as education and employment status can contribute to the health disparities across caregiver groups. Therefore, our age-adjusted estimates remain confounded. Recognizing these limitations, we propose pathways that may guide future hypothesis-generating research. The nature of spousal caregiving itself is distinct, as older couples often share health risks, health behaviors and outcomes (55). Although we do not know the living arrangement of other types of family caregivers, it is probable that spousal caregivers live with their care recipients. This likely translates to around-the-clock care, which may be particularly harmful to the health of caregivers. Moreover, this intense care may create a dual burden for the healthier spouse. Not only are they providing direct care, but they are also assuming the regular household and family responsibilities (e.g., driving, paying bills, household chores) that the spouse needing care can no longer manage (4). Assisting a spouse, who is often of a similar age, with deteriorating health may also signal to the caregivers their own mortality and fail to meet expectations for spousal relationships and responsibilities, underscoring the importance of timing and linked lives. Given the descriptive nature of this study and its limited control for age, these proposed mechanisms are hypothesis-generating, underscoring the need for future longitudinal research using richer data and symmetric operationalization of caregiver status.

While research often groups spousal caregivers and parent caregivers together, our findings underscore the value of analyzing these groups separately (22, 56). In fact, parent caregivers in our study were better off across most sociodemographic and health indicators than the other caregiving groups. Again, selection effects may be at play, as most able siblings in terms of time, location and resources typically assume these caregiving roles. Differences may also be driven partly by unmeasured caregiving burden or demands that differ between parent caregivers and spousal caregivers in this sample.

The finding that spousal caregivers fare worse than parent caregivers map directly onto life course developmental theory in terms of chronological expectations and linked lives. While family caregiving is generally expected in mid-life, caring for aging parents represents an on-time or normative transition that may be less stressful compared to the often unexpected responsibility of providing care to a same-generation spouse (20). Similarly, this pattern can be understood through the concept of linked lives. Providing care to a parent often preserves established intergenerational relationships, although the parent–child dynamic shifts, this transition unfolds predictably over decades and is rarely perceived as an abrupt, life-altering shock. In contrast, spousal caregiving can change marital relationship patterns quickly, leaving spousal caregivers with less time to adjust (13).

Our findings also add to the mixed results linking grandchild caregiving to grandparents’ own wellbeing. Variability in health outcomes of grandchild caregivers depended on the comparison group, pointing to the importance of considering who grandchild caregivers should be compared to in identifying needs, assets and relevance of support services. For example, self-reported good health was the highest and indistinguishable for grandchild and parent caregivers. In fact, we observed consistent wellbeing advantages for parent and grandchild caregivers, possibly stemming from selection factors favoring these caregiver groups. Consistent with the life course developmental framework, perhaps grandchild and parent caregiver roles reflect choices to care and potential satisfaction derived from giving back to parents or paying forward to grandchildren, whereas the spousal and child caregiving roles may involve obligatory caregiving. Because this study is descriptive, future research should analyze other confounding factors, such as caregiver characteristics and intensity, as grandparent caregivers are a diverse group (e.g., raising grandchildren vs. supplementary care).

Patterns across caregiving groups reflect life course developmental disparities in wealth, health and wellbeing. Women were more likely than men to take on caregiving roles in families, particularly grandchild and parent caregivers. This disproportionate contribution underscores the significant impact of unpaid caregiving on women’s later-life health, labor market outcomes, and financial wellbeing (48). Spousal caregivers reported lower income and total household assets than all other caregiver groups, potentially highlighting socioeconomic disadvantage that contributes to greater need for spousal caregiving, worse shared health outcomes, and negative impacts of caregiving on spouses.

This study has limitations. First, we lack access to details such as caregiving intensity (e.g., hours of care), locations (e.g., co-residence), and types of tasks for all caregiving types, which can affect the health outcomes of each caregiver type. In particular, given how the data about spousal caregiving were collected, we were likely to capture high-intensity caregiving for spouses with significant needs. Accounting for this information would facilitate better across-group comparisons. Therefore, future research is needed to identify specific caregiving responsibilities and resources for all caregiver types. Second, we lack participants’ perceptions of their caregiving experiences and the quality of their relationships with the care recipients, which may be relevant to their health. For example, parents over age 50 raising minor children can differ significantly from other caregiver types in terms of role expectations and daily care intensity. Nevertheless, this group represents a critical, understudied mid-life population that warrants further research. Third, we could only account for specific family caregiving relationships, and thus we were unable to assess the extent to which participants cared for others or were helped in their caregiving by others.

Despite the limitations, this study has significant implications for policy, practice, and research. First, our analysis revealed that approximately three out of 10 individuals over age 50 provide care for an older parent, spouse, minor child, or grandchild in the United States. Because caregiving is a dynamic process embedded in interconnected family systems, a health shock or child-rearing transition can trigger a domino effect throughout the family network. This vulnerability is especially concerning given current complex and fragmented caregiver support systems (57). To address this, social and workplace policies should shift from treating caregiving as a temporary, singular burden to recognizing it as a lifelong, systemic reality with the potential for particularly diverse caregiving types in mid-life. This requires the development of integrated family-leave policies and clinical interventions that support the collective resilience of the family system.

Second, this study underscores a critical need to move beyond a “one-size-fits all” support model, highlighting the heterogeneity of family caregivers. Developing effective support requires tailoring interventions to the unique risks of specific caregiving roles. For example, since spousal caregivers are particularly vulnerable to adverse health outcomes, targeted interventions are necessary from healthcare and social work professionals to protect these caregivers before their own health becomes compromised. For caregivers over age 50 who are caring for minor children, the age-adjusted results suggest that report poorer health than those caring for grandchildren and parents. This finding aligns with growing evidence on the stressors of modern parents (29, 58), particularly in the context of increasing parental age, and underscores the need for support, such as access to affordable child care, paid parental leave, social support and reliable parenting advice.

Additionally, with nearly 15% of the family caregivers providing multi-generational support, there is an urgent need for policy shifts such as expanded Family and Medical Leave Act or caregiver payment programs that account for concurrent care demands to alleviate compounding role strain. Future research is needed to examine the processes linked to health and wellbeing among those taking on these complex caregiving roles, including those in traditional (minor child/older parent) and less traditional (grandchild/older parent) caregiving ‘sandwiches’, where varying relationship histories, paid care infrastructures, and cultural expectations uniquely shape caregiver wellbeing. Unlike countries with robust public infrastructure and strong filial piety norms, American families constantly balance the economic and emotional tradeoff between informal family support and away-from-home options. Investigating how these structural and cultural factors interact will be critical to designing interventions that protect caregiver wellbeing across diverse cultural and socio-economic contexts.

## Data Availability

The data analyzed in this study are available from the University of Michigan’s Health and Retirement Study and the RAND Corporation. These data are subject to license restrictions and were used with permission. Information regarding data access can be found through the University of Michigan’s HRS website: https://hrs.isr.umich.edu/.

## Footnotes

1 Due to data limitations, other caregiving relationships such as caring for disabled adult children, siblings, or other relatives were not considered and they were included in the non-caregiver group in our sample. Individuals with missing data on the caregiving variables were also classified as non-caregivers.

2 In the HRS, a proxy respondent (usually a spouse or other family member) answers for those who are unable or unwilling to complete their own interview.

## References

[1] Hunt V. As the US ages, a growing movement aims to care for caregivers. JAMA. (2025) 334:292–4. doi: 10.1001/jama.2025.9658, 40540290

[2] Medina LD Sabo S Vespa J. Living longer: Historical and Projected Life Expectancy in the United States, 1960 to 2060 (Current Population Reports Nos. P25-1145). Suitland: US Department of Commerce U.S. Census Bureau (2020).

[3] Manor S. Being a working grandmother, mother, and daughter at the same time: a “double sandwich” in a four-generation family. J Fam Issues. (2021) 42:324–44. doi: 10.1177/0192513X20921520

[4] Carr D Utz RL. Families in later life: a decade in review. J Marriage Fam. (2020) 82:346–63. doi: 10.1111/jomf.12609, 33633412 PMC7904069

[5] Carlson MJ Wimer C Haskins R. Changing work, changing families, and public policies toward low-income families. RSF Russell Sage Found J Soc Sci. (2022) 8:1–22. doi: 10.7758/rsf.2022.8.5.01

[6] Ansari-Thomas Z. Sandwich caregiving and paid work: differences by caregiving intensity and women’s life stage. Popul Res Policy Rev. (2024) 43:8. doi: 10.1007/s11113-023-09852-5, 40708837 PMC12288679

[7] Ali A Jacocks C. "Family and caregiver dynamics". In: Schechter N Jacocks C Butt L Wegener ST, editors. Motivational Interviewing in Medical Rehabilitation, 1st Edn. New York: Oxford University Press (2024). p. 185–200.

[8] Yousafzai AK. World Health Organization Recommendations on Caregiving Interventions to Support Early Child Development in the First Three Years of Life. Geneva: WHO (2018).

[9] Charenkova J. “Parenting my parents”: perspectives of adult children on assuming and remaining in the caregiver’s role. Front Public Health. (2023) 11:1059006. doi: 10.3389/fpubh.2023.1059006, 36875393 PMC9982148

[10] Pinquart M Sörensen S. Differences between caregivers and noncaregivers in psychological health and physical health: a meta-analysis. Psychol Aging. (2003) 18:250–67. doi: 10.1037/0882-7974.18.2.250, 12825775

[11] Dang S Looijmans A Ferraris G Lamura G Hagedoorn M. Exploring the needs of spousal, adult child, and adult sibling informal caregivers: a mixed-method systematic review. Front Psychol. (2022) 13:832974. doi: 10.3389/fpsyg.2022.832974, 35401295 PMC8992373

[12] Gilligan M Karraker A Jasper A. Linked lives and cumulative inequality: a multigenerational family life course framework. J Fam Theory Rev. (2018) 10:111–25. doi: 10.1111/jftr.12244, 30034068 PMC6051726

[13] Rodrigues R Filipovič Hrast M Kadi S Hurtado Monarres M Hlebec V. Life course pathways into intergenerational caregiving. J Gerontol: Ser B. (2022) 77:1305–14. doi: 10.1093/geronb/gbac024, 35137055 PMC9255941

[14] Bengtson VL Allen KR. "The life course perspective applied to families over time". In: Sourcebook of Family Theories and Methods: A Contextual Approach. Boston: Springer U.S. (1993). p. 469–504.

[15] Seah CWA Yeo TM Hildon ZJ-L Goh Y-SS Chua XHJ Teo JYC . A comprehensive mapping of intergenerational caregiving: a scoping review of dyadic and family caregiving. J Fam Theory Rev. (2026) 1–20. doi: 10.1111/jftr.70067

[16] Pearlin LI Mullan JT Semple SJ Skaff MM. Caregiving and the stress process: an overview of concepts and their measures. The Gerontologist. (1990) 30:583–94. doi: 10.1093/geront/30.5.583, 2276631

[17] Dieker JL Yun SW Weber KL Qualls S. Family conflict over illness beliefs and care strategies: implications for burden in family caregivers. Aging Ment Health. (2024) 28:457–65. doi: 10.1080/13607863.2023.2282683, 37993412

[18] Scott RK Nadorff DK Barnett M Yancura L. Respect your elders: generativity and life satisfaction in caregiving grandparents. Int J Aging Hum Dev. (2023) 96:335–49. doi: 10.1177/00914150221092128, 35404172

[19] Yancura L. Common strengths of various types of family caregiving: applications for research, teaching, and practice in family and consumer sciences. J Fam Consum Sci. (2018) 110:7–13. doi: 10.14307/JFCS110.4.7

[20] Lee C Glei DA Park S Brown CK Weinstein M. When family caregiving happens: life-course timing and health outcomes in later life. J Gerontol B Psychol Sci Soc Sci. (2026) 81:gbag024. doi: 10.1093/geronb/gbag024, 41701143 PMC13075644

[21] AARP & National Alliance for Caregiving. Caregiving in the US 2025. Washington, DC: AARP Public Policy Institute (2025). doi: 10.26419/ppi.00373.001

[22] Schulz R Beach SR Czaja SJ Martire LM Monin JK. Family caregiving for older adults. Annu Rev Psychol. (2020) 71:635–59. doi: 10.1146/annurev-psych-010419-050754, 31905111 PMC7291827

[23] Doyle KL Toepfer M Bradfield AF Noffke A Ausderau KK Andreae S . Systematic review of exercise for caregiver–care recipient dyads: what is best for spousal caregivers—exercising together or not at all? The Gerontologist. (2021) 61:e283–301. doi: 10.1093/geront/gnaa043, 32614050 PMC8361501

[24] Bueno MV Chase J-AD. Gender differences in adverse psychosocial outcomes among family caregivers: a systematic review. West J Nurs Res. (2023) 45:78–92. doi: 10.1177/01939459221099672, 35614567

[25] Pinquart M Sörensen S. Spouses, adult children, and children-in-law as caregivers of older adults: a meta-analytic comparison. Psychol Aging. (2011) 26:1–14. doi: 10.1037/a0021863, 21417538 PMC4449135

[26] del-Pino-Casado R Priego-Cubero E López-Martínez C Orgeta V. Subjective caregiver burden and anxiety in informal caregivers: a systematic review and meta-analysis. PLoS One. (2021) 16:e0247143. doi: 10.1371/journal.pone.0247143, 33647035 PMC7920375

[27] Lysons J Jadva V. The psychosocial outcomes of older parenthood in early to mid-childhood: a mini-review. Hum Reprod. (2023) 38:1028–35. doi: 10.1093/humrep/dead070, 37036943 PMC10233307

[28] Nelson SK Kushlev K English T Dunn EW Lyubomirsky S. In defense of parenthood: children are associated with more joy than misery. Psychol Sci. (2013) 24:3–10. doi: 10.1177/0956797612447798, 23201970

[29] Van Wijk D Billari FC. Fertility postponement, economic uncertainty, and the increasing income prerequisites of parenthood. Popul Dev Rev. (2024) 50:287–322. doi: 10.1111/padr.12624

[30] Crowther MR. Supporting Grandparents Raising Grandchildren (SGRG) Act Initial Report to Congress. Washington: Administration for Community Living (ACL) (2021).

[31] Arpino B Gómez-León M. Consequences on depression of combining grandparental childcare with other caregiving roles. Aging Ment Health. (2020) 24:1263–70. doi: 10.1080/13607863.2019.1584788, 30870002

[32] Leonhardt M. The unsung heroes of the American economy: Grandmothers (2023). Available online at: https://fortune.com/2023/03/08/the-unsung-heroes-of-the-american-economy-grandmothers/ (Accessed March 12, 2026).

[33] Kerman S Amerikaner L Houghton A. Powering Families: The Essential Role of Grandparents in Care, Connection and Support. Washington, DC: AARP Research (2026).

[34] Danielsbacka M Křenková L Tanskanen AO. Grandparenting, health, and well-being: a systematic literature review. Eur J Ageing. (2022) 19:341–68. doi: 10.1007/s10433-021-00674-y, 36052183 PMC9424377

[35] Hayslip B Fruhauf CA Dolbin-MacNab ML. Grandparents raising grandchildren: what have we learned over the past decade? The Gerontologist. (2019) 59:e152–63. doi: 10.1093/geront/gnx106, 28666363

[36] Overton WF. "Life-span development: concepts and issues". In: Lerner RM Lamb ME Freund AM, editors. The Handbook of Life-Span Development, 1st EdnWiley Hoboken: John Wiley & Sons. (2010)

[37] Baltes PB Reese HW Lipsitt LP. Life-span Developmental Psychology. Annu Rev Psychol. (1980) 31:65–110. doi: 10.1146/annurev.ps.31.020180.000433, 7362217

[38] Cohen SA Greaney ML. Aging in rural communities. Curr Epidemiol Rep. (2022) 10:1–16. doi: 10.1007/s40471-022-00313-9, 36404874 PMC9644394

[39] World Health Organization. Ageing and Health. Geneva: WHO (2025).

[40] Aldwin CM Yancura L Lee H. "Stress, coping, and aging". In: Handbook of the Psychology of Aging. San Diego, CA: Academic Press (2021). p. 275–86.

[41] Abdollahpour I Nedjat S Noroozian M Salimi Y Majdzadeh R. Caregiver burden: the strongest predictor of self-rated health in caregivers of patients with dementia. J Geriatr Psychiatry Neurol. (2014) 27:172–80. doi: 10.1177/0891988714524627, 24614200

[42] Dellafiore F Arrigoni C Nania T Caruso R Baroni I Vangone I . The impact of COVID-19 pandemic on family caregivers’ mental health: a rapid systematic review of the current evidence: COVID-19 and family caregivers’ mental health. Acta Biomed Atenei Parm. (2022) 93:e2022154. doi: 10.23750/abm.v93iS2.12979, 35545977 PMC9534216

[43] Zan H Shin SH. The positive impact of informal spousal caregiving on the physical activity of older adults. Front Public Health. (2022) 10:977846. doi: 10.3389/fpubh.2022.977846, 36589971 PMC9800888

[44] Drentea P Pavela G Tian L Locher JL. Grandparenting and physical activity. J Aging Health. (2025) 37:543–54. doi: 10.1177/08982643241273197, 39180377 PMC12933441

[45] Sonnega A. Using Health and Retirement Study data: A guide for new Users. Ann Arbor: Survey Research Center, Institute for Social Research, University of Michigan (2025).

[46] Bugliari D Carroll J Hayden O Hayes J Hurd MD Lee S . RAND HRS Longitudinal File 2020 (V2) Documentation. Santa Monica: RAND Corporation (2024).

[47] Choi NG Burr JA Mutchler JE Caro FG. Formal and informal volunteer activity and spousal caregiving among older adults. Res Aging. (2007) 29:99–124. doi: 10.1177/0164027506296759

[48] Lee Y Tang F. More caregiving, less working: caregiving roles and gender difference. J Appl Gerontol. (2015) 34:465–83. doi: 10.1177/0733464813508649, 24652908

[49] Hupin D Roche F Gremeaux V Chatard J-C Oriol M Gaspoz J-M . Even a low-dose of moderate-to-vigorous physical activity reduces mortality by 22% in adults aged ≥60 years: a systematic review and meta-analysis. Br J Sports Med. (2015) 49:1262–7. doi: 10.1136/bjsports-2014-094306, 26238869

[50] Saint-Maurice PF Troiano RP Berrigan D Kraus WE Matthews CE. Volume of light versus moderate-to-vigorous physical activity: similar benefits for all-cause mortality? J Am Heart Assoc. (2018) 7:e008815. doi: 10.1161/JAHA.118.008815, 29610219 PMC5907606

[51] StataCorp. Stata 19 Base Reference Manual. College Station: Stata Press (2025).

[52] Lei L Leggett AN Maust DT. A national profile of sandwich generation caregivers providing care to both older adults and children. J Am Geriatr Soc. (2023) 71:799–809. doi: 10.1111/jgs.18138, 36427297 PMC10023280

[53] Pierret CR. The sandwich generation: women caring for parents and children. Mon Lab Rev. (2006) 129:3. Available online at: https://www.bls.gov/opub/mlr/2006/09/art1full.pdf

[54] Cohen J. Statistical power Analysis for the Behavioral Sciences. 2nd ed. Hillsdale, NJ: Lawrence Erlbaum Associates Inc. (1988).

[55] Meyler D Stimpson JP Peek MK. Health concordance within couples: a systematic review. Soc Sci Med. (2007) 64:2297–310. doi: 10.1016/j.socscimed.2007.02.007, 17374552

[56] Kunicki ZJ Gaudiano BA Miller IW Tremont G Salloway S Darling E . Differences in burden severity in adult-child family caregivers and spousal caregivers of persons with dementia. J Gerontol Soc Work. (2021) 64:518–32. doi: 10.1080/01634372.2021.1912242, 33820479

[57] Charles L Brémault-Phillips S Parmar J Johnson M Sacrey L-A. Understanding how to support family caregivers of seniors with complex needs. Can Geriatr J. (2017) 20:75–84. doi: 10.5770/cgj.20.252, 28690707 PMC5495539

[58] The US Surgeon General. Parents Under Pressure: The US Surgeon General’s Advisory on the Mental Health & Well-Being of Parents. New York: National Institutes of Health (NIH) (2024).

